# Successful Inhibition of Tumor Development by Specific Class-3 Semaphorins Is Associated with Expression of Appropriate Semaphorin Receptors by Tumor Cells

**DOI:** 10.1371/journal.pone.0003287

**Published:** 2008-09-26

**Authors:** Boaz Kigel, Asya Varshavsky, Ofra Kessler, Gera Neufeld

**Affiliations:** Cancer Research and Vascular Biology Center, The Bruce Rappaport Faculty of Medicine, Technion, Israel Institute of Technology, Haifa, Israel; University of Minnesota, United States of America

## Abstract

The class-3 semaphorins (sema3s) include seven family members. Six of them bind to neuropilin-1 (np1) or neuropilin-2 (np2) receptors or to both, while the seventh, sema3E, binds to the plexin-D1 receptor. Sema3B and sema3F were previously characterized as tumor suppressors and as inhibitors of tumor angiogenesis. To determine if additional class-3 semaphorins such as sema3A, sema3D, sema3E and sema3G possess anti-angiogenic and anti-tumorigenic properties, we expressed the recombinant full length semaphorins in four different tumorigenic cell lines expressing different combinations of class-3 semaphorin receptors. We show for the first time that sema3A, sema3D, sema3E and sema3G can function as potent anti-tumorigenic agents. All the semaphorins we examined were also able to reduce the concentration of tumor associated blood vessels although the potencies of the anti-angiogenic effects varied depending on the tumor cell type. Surprisingly, there was little correlation between the ability to inhibit tumor angiogenesis and their anti-tumorigenic activity. None of the semaphorins inhibited the adhesion of the tumor cells to plastic or fibronectin nor did they modulate the proliferation of tumor cells cultured in cell culture dishes. However, various semaphorins were able to inhibit the formation of soft agar colonies from tumor cells expressing appropriate semaphorin receptors, although in this case too the inhibitory effect was not always correlated with the anti-tumorigenic effect. In contrast, the anti-tumorigenic effect of each of the semaphorins correlated very well with tumor cell expression of specific signal transducing receptors for particular semaphorins. This correlation was not broken even in cases in which the tumor cells expressed significant concentrations of endogenous semaphorins. Our results suggest that combinations of different class-3 semaphorins may be more effective than single semaphorins in cases in which tumor cells express more than one type of semaphorin receptors.

## Introduction

The neuropilin-1 (np1) and the neuropilin-2 (np2) receptors were originally characterized as receptors for axon guidance factors of the class-3 semaphorin (sema3) family [1]. It was subsequently realized that neuropilins are also expressed by endothelial cells and by many types of cancer cells [2]. Neuropilins function in addition as receptors for several angiogenic factors including heparin binding forms of VEGF and hepatocyte growth factor/scatter factor (HGF/SF) and enhance their pro-angiogenic activity [3]–[6]. These studies indicate that neuropilins may be targets for anti-angiogenic therapy. Indeed, antibodies directed against np1 and np2 were recently found to inhibit tumor progression [7], [8].

Most of the sema3s, with the exception of sema3E which binds to Plexin-D1 (plexD1) [9], bind to one of the two neuropilins or to both. Neuropilins form functional semaphorin receptors by associating with members of the plexin receptor family in which neuropilins bind semaphorins and the plexins function as the signal transducing elements [10], [11]. The four type-A plexins as well as plexD1 were found to participate in neuropilin mediated signal transduction [10]–[12]. The semaphorins sema3B and sema3F were characterized as tumor suppressor genes indicating that additional semaphorins may also possess anti-tumorigenic properties [13], [14]. The identification of neuropilins in endothelial cells suggested that class-3 semaphorins may also regulate angiogenesis. Indeed, the np2 agonist sema3F functions as a repellant of endothelial cells. It also induces apoptosis of endothelial cells, and inhibits tumor angiogenesis and tumor progression [15], [16]. The np1 agonist sema3A also inhibits angiogenesis [17] but it is as yet unknown whether it can inhibit tumor angiogenesis and tumor progression. Likewise, the plexD1 agonist sema3E was found to inhibit the invasion of blood vessels into somites during embryonic development [9] suggesting that sema3E too may function as an anti-angiogenic agent.

The expression of neuropilins and plexins by many types of tumor cells indicates that semaphorins may be able to affect tumor cells directly. Indeed, sema3F and sema3B have been found to inhibit the adhesion, migration and proliferation of several types of lung cancer derived tumor cells [13], [14], [16], [18]. It follows that semaphorins such as sema3F probably inhibit angiogenesis and tumor cell proliferation simultaneously and may also affect in addition the behavior of additional types of stromal cells. However, it is unclear which of these mechanisms is the primary mechanism used by semaphorins such as sema3F to inhibit tumor development. It is also unclear whether additional sema3s possess anti-angiogenic and anti-tumorigenic properties. We report that four additional class-3 semaphorins which have not yet been found to possess anti-tumorigenic properties, sema3A, sema3D, sema3E, and sema3G possess anti-tumorigenic properties. Furthermore, all these semaphorins with the exception of sema3E strongly reduce the density of blood vessels in tumors. However, we find that inhibition of tumor development by class-3 semaphorins is strongly correlated with the expression of appropriate semaphorin receptors by the tumor cells and that there is a much poorer correlation between their ability to inhibit angiogenesis and their effects on tumor development.

## Materials and Methods

### Materials

Antibodies against β-actin and myc and FLAG epitope tags, as well as chemicals were from Sigma (St. Louis, MI). Media and sera for cell culture were from Biological-Industries Inc. (Kibbutz Beth-Haemek, Israel). Fugene-6 was from Roche Ltd (Switzerland). Antibodies against np1 and np2 were purchased from Santa-Cruz inc. (San-Diego, CA). The cDNAs encoding different semaphorins were subcloned into the NSPI-CMV-MCS-myc-His lentiviral expression vector containing SV40 promoter driving Puromycin selection marker. This vector was kindly given to us by Dr. Aaronson (Mount Sinai Hospital, NY). Partial cDNAs encoding sema3E were kindly given to us by Dr. Claus Christensen (Institute of cancer biology, Copenhagen, Denmark). Antibodies against CD-31 were from BD biosciences Pharmingen. The cDNA's encoding sema3F and sema3A were donated by Dr. Mark Tessier-Lavigne (University of California, San Francisco, CA) and by Drs. David Ginty and Alex Kolodkin (Johns Hopkins University, Baltimore, MD). The PerfectPure RNA reverse PCR kit was from 5-Prime (Gaithersburg, MD).

### Primers

The following specific primers were used to follow the expression of different plexins and semaphorins in the cancer cell lines. Plexin-A1: 5′-ctgctggtcatcgtggctgtgct 5′-gggcccttctccatctgctgcttga. Plexin-A2: 5′-gtgcccaccaactgtgcctgtcctg 5′-tcagcgatgatgtattcccctggga. Plexin-A3: 5′-tcttgctctcgaggttcttct 5′-acatgccaagtgatcaacgac. Plexin-A4: 5′-acggtccatcccaacaatatc 5′-ccacgccagcaaccttgacat. Plexin-D1: 5′-gtccatctaccagggcttct 5′-ctggatgtaggactcggtga. Sema3A: 5′-aacgggggcttttcatcc 5′-cccttctcacatcactcatgct. Sema3D: 5′-ggctgctgaggatcgaaggac 5′-atgtgtgtggaactggagca. Sema3E: 5′-gggttacttactggagctttgg 5′-gtcatgctcagtgcggatatg. Sema3F: 5′-gtgctgcccaaggatgacca 5′-cttgttggcattggagttgaacc. Sema3G: 5′-aacgcagagctggccgagga 5′-ccggacccacctgcta. Actin: 5′-tgacggggtcacccacactgtgcccatcta 5′-ctagaagcattgcggtggacgatggaggg.

### Expression plasmids

All the class-3 semaphorin cDNAs were sub-cloned into the NSPI-CMV-myc-his lentiviral expression vector. The sema3G cDNA was cloned from HUVEC mRNA using RT-PCR. The sema3D cDNA was cloned using RT-PCR from HUVEC cells treated with 30 ng/ml of VEGF for 6 hours. cDNA's containing the myc epitope tag were added in frame upstream to the stop codon of sema3D, sema3E, sema3F and sema3G. A FLAG epitope tag was added upstream to the stop codon of sema3A as described [19].

### Generation of recombinant lentiviruses and letiviral mediated infection of cells

HEK293-T cells were seeded in 100 mm tissue culture dishes (2.5×10^6^ cells/dish). A day after seeding, the cells were co-transfected with the appropriate lentiviral expression plasmid (8µg), with the packaging vector pCMVdR8.91 (5 µg), and with a plasmid encoding the vesicular stomatitis virus coat envelope pMD2-VSVG (2 µg) using Fugene-6 according to the instructions of the vendor. Conditioned medium containing infective lentiviral particles was collected 48 hours and 72 hours post transfection. Following addition of polybrene (8 µg/ml) to the conditioned medium it was incubated 8 hours with target cells.

### Cell lines

Mycoplasma free MDA-MB-231, MDA-MB-435, MDA-MB-468 and MCF7 cancer cells were obtained from the ATCC. The cells were cultured in DMEM containing 4.5 mg/ml glucose supplemented with 10% FCS and antibiotics. HUVEC, PAE, HEK293 and HEK293-T cells were cultured as previously described [15]. HUVEC were used between passages 3–7.

### Animal experiments

All the animal experiments were approved by the institutional committee for animal studies according to the NIH guidelines (license IL-095-10-2007).

### *In-vivo* tumor formation assays

Cells expressing semaphorins or control cells infected with empty lentiviral vectors were implanted (5×10^6^/mouse) into the mammary fat pads of 4–6 week old balb\c nu/nu female mice (Harlan laboratories). In most experiments we used groups of 9 animals/experiment. The tumors were measured twice a week using a caliper. The tumor volume (V) was determined using the formula, V = 0.52×A^2^×B in which A is the short diameter and B the long. When MDA-MB-231 tumors reached an average volume of 200–300 mm^3^, they were excised and weighted. Each experiment was repeated at least twice. Estrogen pellets were used in experiments in which the development of tumors from MCF-7 cells was determined as previously described [20].

### Immunohistochemistry

Tumors were embedded in OCT and frozen in 2-methylbutane cooled by liquid nitrogen. They were then sectioned into 30 µm thick sections using a cryostat. Sections were blocked with cold acetone, reacted with an antibody directed against the endothelial marker CD-31, counterstained with hematoxilin and photographed. Eight different microscopic fields derived from different sections of three different tumors were photographed. These photographs were taken from areas in which the density of blood vessels was highest (hot spot method) [21], [22]. The area of the blood vessels in fields of equal area was quantified using the Image Pro Plus software.

### Western Blots

Cell lysates were prepared and the concentration of protein determined as previously described [19]. To determine the concentration of secreted sema3s in conditioned mediums of the various cell lines, cells were seeded in 12 well dishes at a concentration of 2×10^5^ cells/well. The cells were incubated for 48 hours in 0.4 ml of serum-free medium. Aliquots of equal volume were examined using western blot analysis for the presence of sema3s using antibodies directed against the appropriate tags as previously described [19] and the densitometry analysis was preformed using MultiGauge software (FUJIFILM). The expressed semaphorins did not affect the proliferation rate or the survival of the different semaphorin producing cells (data not shown).

### Proliferation assays

Tumor cells (10^4^ cells/well) were seeded in triplicate in 24 well dishes. Adherent cells were trypsinized and counted every 24 hours for 4 days, using a coulter counter.

### Adhesion assays

In cell adhesion experiments we used uncoated 12 well cell culture dishes as well as non-adhesive 12 well dishes coated with fibronectin (5 µl/ml). Tumor cells (10^5^ cells/well) were seeded in triplicates in growth media. The cells were washed twice with PBS, trypsinized to release adherent cells, and counted with a coulter counter. The cells were counted 5, 10, 20 and 45 minutes after they were seeded. The percentage of adherent cells relative to the number of seeded cells was then calculated and plotted. The time required for the adherence of 50% of the seeded cells was used as a measure to compare the adhesive properties of control and semaphorin expressing cells.

### Endothelial cells repulsion assay

Cell repulsion assays were performed essentially as previously described [19].

### Soft-agar colony formation assay

A layer of agar containing 2 ml of 0.5% low melting agar (Bio-Rad) dissolved in growth media was poured into wells of a 6 well cell culture dish and allowed to set at 4°C for 20 minutes. A second layer (1ml) containing 0.3% of low melting agar dissolved in growth media containing cells (3×10^3^ cells/ml) was placed on top of the first layer and allowed to set at 4°C for 20 minutes. Growth medium (2 ml) was added on top of the second layer and the cells were incubated in a humidified incubator at 37°C for 21 days. Medium was changed twice a week. At the end of the experiment, colonies were stained for 1 hr with 0.005% crystal violet, and incubated with PBS overnight to remove excess crystal violet. The colonies were photographed and colonies with at least one diameter of 150 µm within photographic fields were chosen. The Image-pro morphometric software was then used to measure the area of each of these colonies. Their average area and statistics were then performed using the Microsoft excel software.

### Statistical analysis

Statistical analysis was performed using the unpaired data with unequal variance student's T-test. Error bars represent the standard error of the mean. Statistical significance is presented in the following manner: *p<0.05, **p<0.01 and ***p<0.001.

## Results

### Expression patterns of class-3 semaphorins and class-3 semaphorin receptors in different tumorigenic cell lines

**Figure 1:**
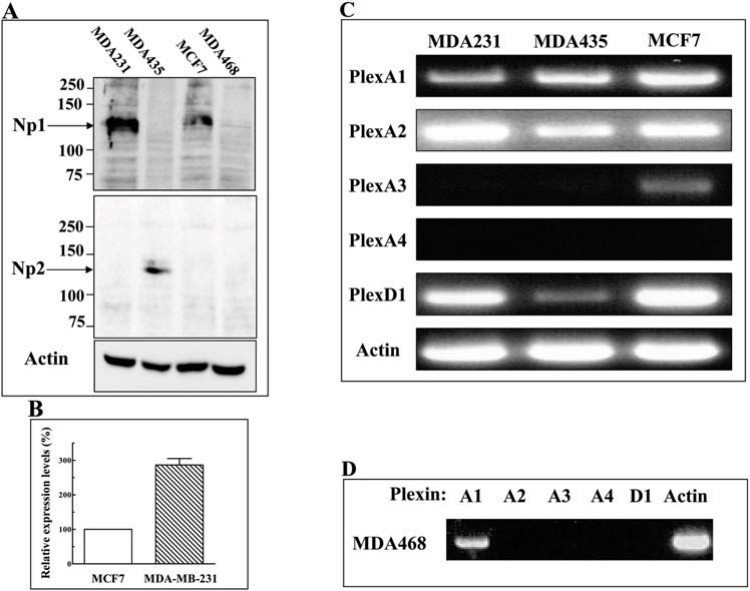
Expression of semaphorin receptors in different tumorigenic cell lines. (A) Cells were grown to 80% confluence and lysed. Equal amounts of protein were loaded and separated on SDS/PAGE gels and subsequently blotted on nitrocellulose filters. Western blot analysis of np1 and np2 was performed as described in materials and methods. (B) Densitometric analysis of three independent experiments showing the relative expression levels of np1 in MDA-MB-231 and MCF-7 cells was preformed using the MultiGauge software. The average expression level of np1 in the MCF-7 cells was taken as 100% and the average expression level of np1 in the MDA-MB-231 cells was compared to the average expression level in MCF-7 cells. (C, D) Reverse PCR analysis of the expression of mRNA's encoding plexA1-A4 and plexD1 was performed according to the instruction of the PerfectPure kit using primer pairs specific to the different plexins as described.

Semaphorins may affect the development of tumors by directly affecting the behavior of tumor cells or indirectly by affecting angiogenesis or the behavior of stromal cells. To find out if the class-3 semaphorins sema3A, sema3D, sema3E, sema3F and sema3G can inhibit the formation of tumors from cancer cells by directly influencing tumor cell behavior, we first determined the expression patterns of known sema3 receptors in different cancer cell lines. We found that MDA-MB-231 breast cancer derived cells express predominantly np1 [4], a receptor for sema3A and sema3D, but very little np2 if at all. MDA-MB-435 melanoma cells express predominantly np2, a receptor for sema3F and sema3G and very little if any np1 (Fig. 1A). MCF-7 breast cancer cells express np1 but not np2. The concentration of np1 in MCF-7 cells is about three folds lower as compared to MDA-MB-231 cells (Fig. 1, A–B). MDA-MB-468 breast cancer cells differ from the other cell lines since they do not express neuropilins (Fig. 1A).

Because of their short intracellular domains neuropilins do not transduce sema3 signals independently but form complexes with plexins in which the plexins serve as the signal transducing elements [10], [11]. Good antibodies to plexins are not yet readily available so we compared the expression of mRNA encoding various plexins known to transduce class-3 semaphorins signals qualitatively using RT-PCR as an indication for possible protein expression. All four cell lines expressed the plexA1 mRNA and all but the MDA-MB-468 cells also expressed the plexA2 mRNA. None of these cell lines expressed the plexA4 mRNA and only the MCF-7 cells expressed the plexA3 mRNA (Fig. 1, B–C). The mRNA encoding the sema3E receptor PlexD1 was expressed in MDA-MB-231 and MCF-7 cells while MDA-MB-435 cells seem to express a lower concentration of PlexD1 mRNA (Fig. 1C) and MDA-MB-468 cells did not express plexD1 mRNA at all (Fig. 1, C–D). The expression of the neuropilins and of the mRNA encoding the sema3E receptor PlexD1 in the various tumor cells was not altered significantly as a result of the expression of the various recombinant semaphorins ( Figs. S1 and S2).

### The effects of different sema3s on the development of tumors from cancer cells

**Figure 2:**
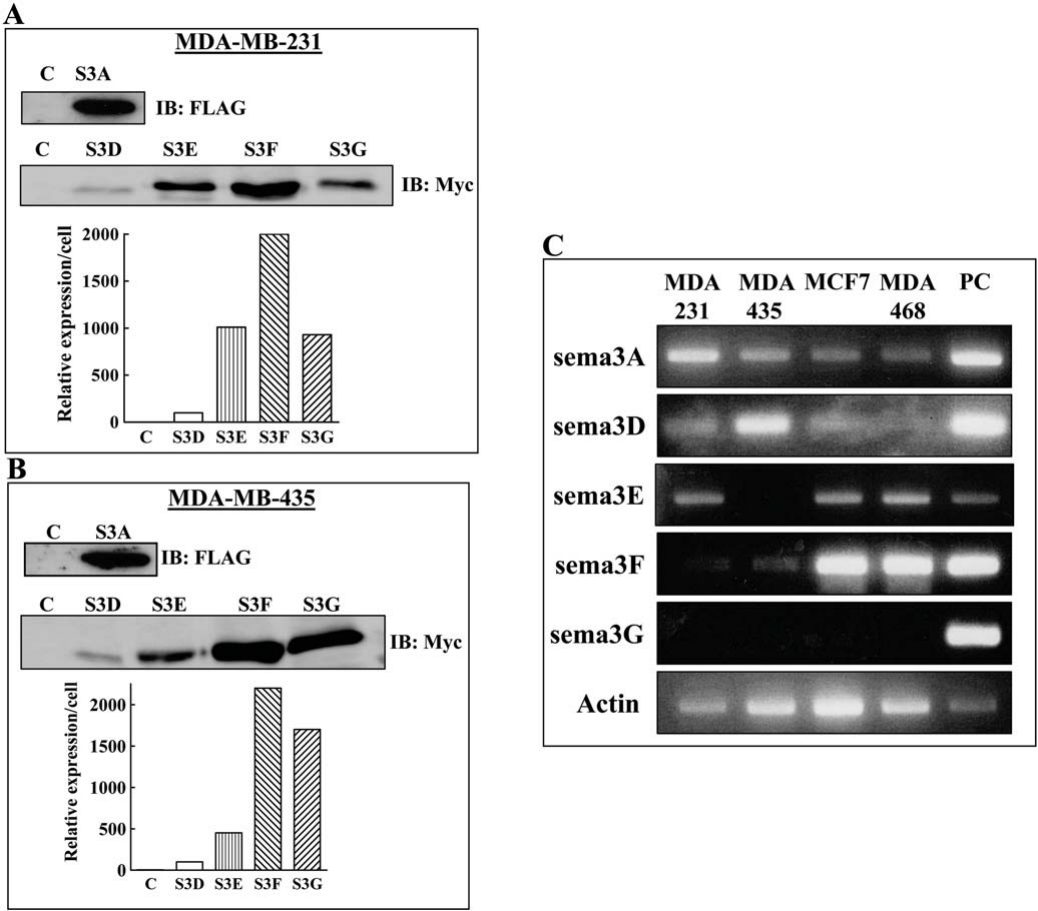
Determination of the relative concentrations of recombinant class-3 semaphorins secreted into the conditioned medium of MDA-MB-231 and MDA-MB-435 cells. Lentiviruses containing the full length cDNAs encoding five semaphorins and a puromycin resistance cassette or empty control lentiviruses were used to infect MDA-MB-231 and MDA-MB-435 cells. Sema3A has a flag epitope tag, whereas the rest of the semaphorins were labeled with a myc epitope tag. (A, B) Western blot analysis of equal aliquots of conditioned medium derived from equal numbers of MDA-MB-231 and MDA-MB-435 cells expressing the different sema3s. The expression levels of all the myc tagged semaphorins was quantified as described in materials and methods. (C) Reverse PCR analysis of endogenous mRNA's encoding sema3A, sema3D, sema3E, sema3F and sema3G expression was performed according to the instruction of the PerfectPure kit using primer pairs specific to the different semaphorins as described. MDA-MB-231 cells over-expressing the different recombinant semaphorins were used as positive controls.

To find out if sema3A, sema3D, sema3E, sema3F or sema3G over-expression can affect the development of tumors from different tumorigenic cell lines, we expressed the full length cDNAs encoding the five semaphorins or an empty control vector in the tumor cells using a lentiviral expression vector that confers resistance to puromycin. Pools of infected cells were examined for semaphorin expression following puromycin selection using antibodies directed against epitope tags incorporated into the recombinant semaphorins. The expression levels of the recombinant semaphorins seemed to differ in correlation with the type of the recombinant semaphorin and much less so in correlation with the cell type in which they were expressed. Thus, the concentration of recombinant sema3D found in the conditioned medium of either MDA-MB-231 breast cancer cells or in MDA-MB-435 melanoma cells was significantly lower than the concentrations of sema3F or sema3G (Fig. 2, A–B). It was not possible to effectively compare the concentrations of the endogenous semaphorins produced by the tumor cells with the concentrations of the recombinant semaphorins expressed in each of the cell types due to the lack of suitable highly specific antibodies directed against the various semaphorins. However, from reverse transcription followed by PCR (RT-PCR) experiments it is clear that the various tumor cells we studied also express various endogenous mRNA's encoding class-3 semaphorins suggesting that these cells may produce combinations of endogenous semaphorins. Thus, MDA-MB-435 cells express mRNA encoding sema3D while MDA-MB-231 cells express sema3A and sema3E mRNA while MCF-7 and MDA-MB-468 cells express mRNA encoding sema3F (Fig. 2C).

**Figure 3:**
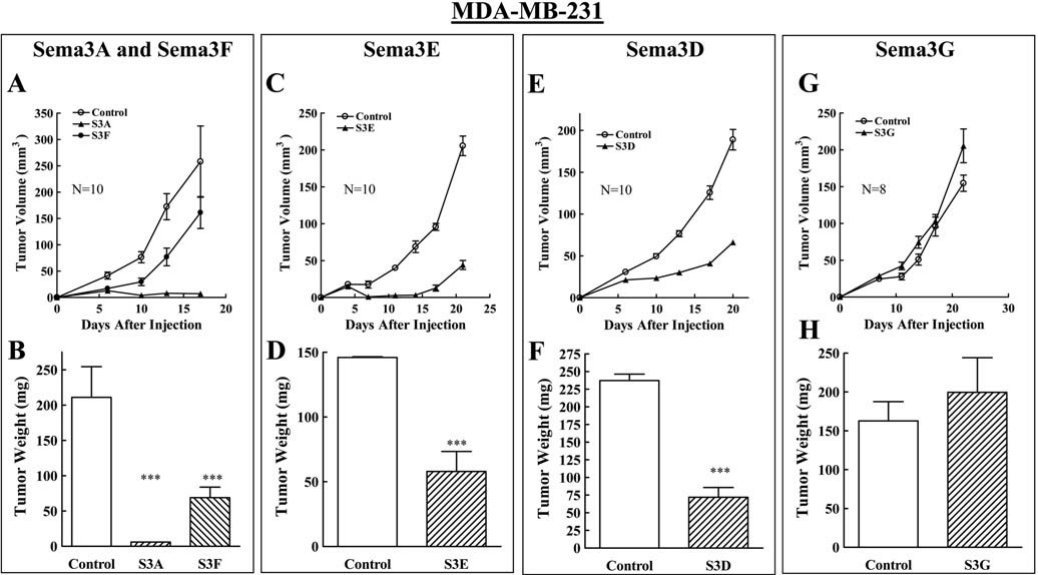
The effect of the expression of different sema3s on the development of tumors from MDA-MB-231 cells. MDA-MB-231 cells infected with control lentivirus or infected with lentiviruses directing expression of five different semaphorins were implanted in the mammary fat pads of balb\c nu/nu mice as described. (A, C, E, G) The average volume of the developing tumors was measured as a function of time after implantation as described in materials and methods. (B, D, E, F) The average weight of the tumors at the end of the experiment was determined as described in materials and methods.

Sema3s contain conserved cleavage sites for furin like pro-protein convertases[23]. In the case of sema3E the cleaved product was reported to possess pro-metastatic properties [24]. However, the degree of cleavage of the recombinant semaphorins produced by MDA-MB-231 cells or by MDA-MB-435 cells did not exceed 15% of the total amount of semaphorin found in the conditioned medium and in the case of sema3E was almost undetectable (data not shown). The cells were subsequently implanted in mammary fat pads of immune deficient mice, and allowed to form tumors. All these semaphorins were relatively efficiently expressed in MDA-MB-231 cells although there were substantial differences in the expression levels obtained with different semaphorins (Fig. 2A). Expression of the np1 agonist sema3A [25] inhibited almost completely the development of tumors from these cells (Fig. 3, A–B and Fig. S3, A). Sema3D, an agonist for both np1 and np2 [26], inhibited tumor formation completely in one experiment (data not shown) and in another experiment inhibited strongly though not completely tumor development even though it was not as highly expressed as the other semaphorins (Fig. 3, E–F). In contrast, the np2 agonist sema3G [27] was unable to inhibit tumor development from these cells, although it was highly expressed as compared to sema3D (Fig. 3, G–H). The np2 agonist sema3F on the other hand, inhibited significantly the development of tumors despite the lack of np2 receptors in MDA-MB-231 cells (Fig. 3, A–B). The tumors that developed from sema3F expressing MDA-MB-231 cells (Fig. 3, A–B) looked less bloody than the control tumors suggesting that sema3F may inhibit tumor angiogenesis (Fig. S3, A). Expression of the PlexD1 agonist sema3E [9] also inhibited significantly the development of tumors from these cells but the resulting tumors did not look starved of blood vessels (Fig. 3, C–D, and Fig. S3, B).

**Figure 4:**
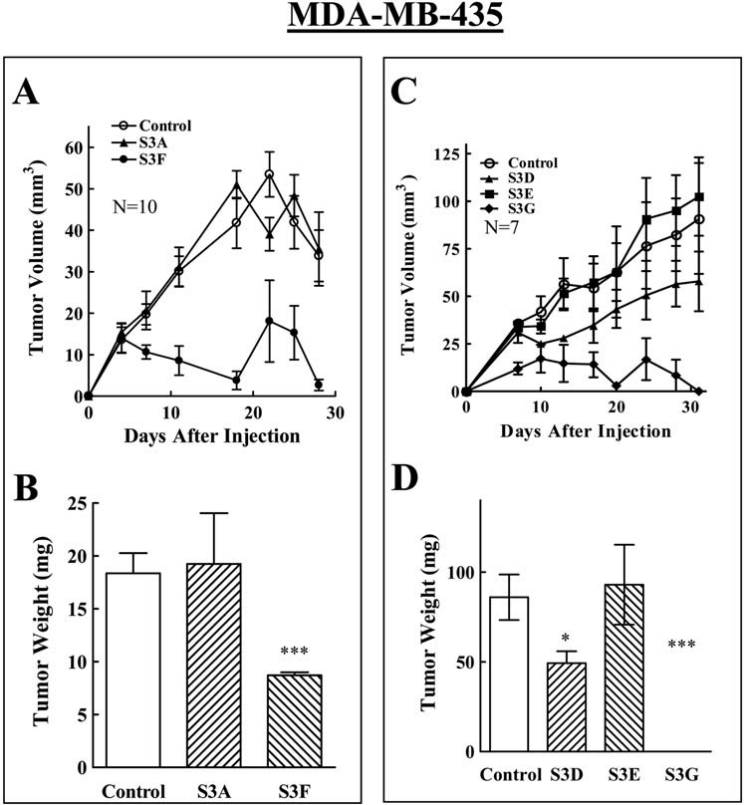
The effect of the expression of different sema3s on the development of tumors from MDA-MB-435 cells. MDA-MB-435 cells infected with control lentivirus or infected with lentiviruses directing expression of five different semaphorins were implanted in the mammary fat pads of balb\c nu/nu mice as described. (A, C) The average volume of the developing tumors was measured as described in materials and methods. (B, D) The average weight of the tumors at the end of the experiment was determined as described in materials and methods.

A different picture emerged when the effects of these sema3s on the development of tumors from MDA-MB-435 cells were examined. Control cells developed into small tumors that slowed when they reached an average volume of 50–100 mm^3^ (Fig. 3, A–B). Expression of the np2 agonists sema3F and sema3G strongly inhibited the development of tumors from these cells (Fig. 4, A–D). In contrast, expression of sema3A did not inhibit tumor development (Fig. 4, A–B), while sema3D which binds to both neuropilins [26], significantly inhibited tumor development from the MDA-MB-435 cells though less potently than sema3G (Fig. 4, C–D) which may be due to the lower expression levels obtained with sema3D in these cells (Fig. 2B). MDA-MB-435 cells also express the PlexD1 mRNA, although at lower levels than MDA-MB-231 cells (Fig. 1C). Expression of sema3E did not inhibit the formation of tumors from the MDA-MB-435 cells (Fig. 4 C–D). This was probably not due to cleavage by furin like pro-protein convertases since less than 5% of the sema3E found in the conditioned medium of these cells was cleaved (data not shown).

**Figure 5:**
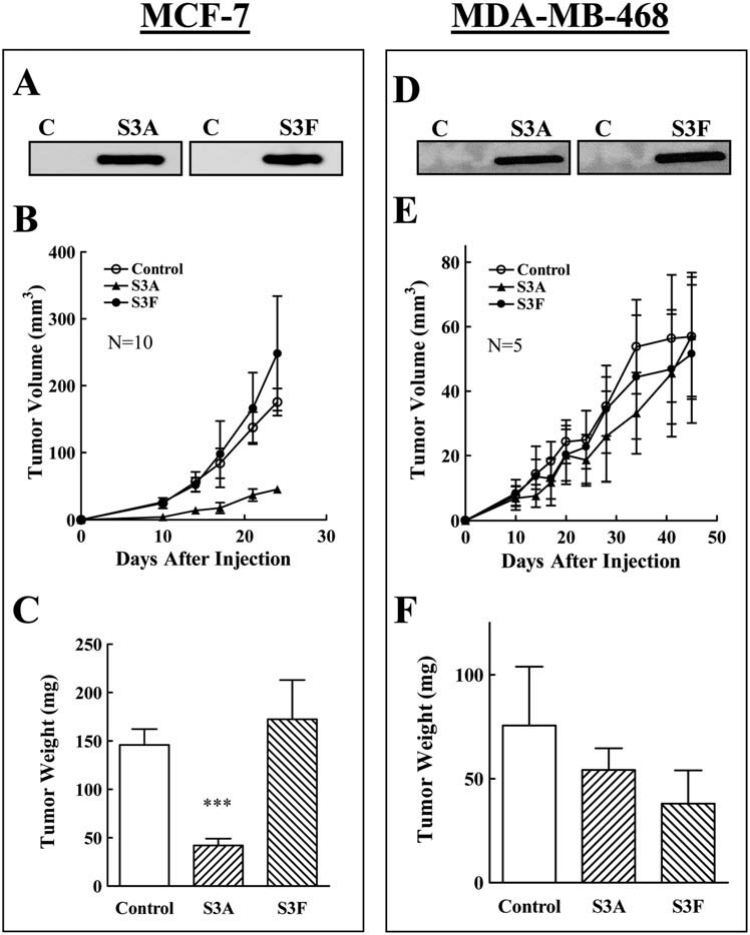
The effect of the expression of different sema3s on the development of tumors from MCF-7 and MDA-MB-468 cells. MCF-7 and MDA-MB-468 cells infected with control lentivirus or infected with lentiviruses directing expression of five different semaphorins were implanted in the mammary fat pads of balb\c nu/nu mice as described. (A, D) Western blot analysis of aliquots of conditioned medium derived from equal numbers of cells infected with empty lentiviruses (C) or with different sema3s (s3X) as indicated. (B, E) The average volume of the developing tumors was measured as described in materials and methods. (C, F) The average weight of the tumors at the end of the experiment was determined as described in materials and methods.

We also determined whether expression of sema3A and sema3F, the best studied np1 and np2 agonists respectively, inhibits tumor development from the non-metastatic, estrogen dependent, np1 expressing MCF-7 cells. Expression of sema3A inhibited significantly tumor development while expression of sema3F did not (Fig. 5, B–C). Taken together, these results suggested that the sema3s ability to inhibit tumor formation from a given cancer cell type depends primarily on the identity of the semaphorin receptors expressed by the tumor cells, suggesting that sema3s should not be able to inhibit the formation of tumors from cancer cells that do not express sema3 receptors (Table 1).

**Table 1:** Summary of the results.

Cell line	Expression of recombinant semaphorin	Known binding receptors	Expression of semaphorin receptors in tumor cells			Inhibition of tumor development	Inhibition of angiogenesis	Inhibition of soft agar colony formation
			NP1	NP2	PlexD1**			
MDA-MB-231	S3A	NP1	+++		+++	✓	N.D.*	✓
	S3D	NP1, NP2				✓	✓	✓
	S3E	PlexD1				✓	NO	NO
	S3F	NP1, NP2				***✓	✓	✓
	S3G	NP2				NO	✓	NO
MDA-MB-435	S3A	NP1		+++	+	NO	✓	NO
	S3D	NP1, NP2				✓	✓	✓
	S3E	PlexD1				NO	✓	✓
	S3F	NP1, NP2				✓	N.D.*	NO
	S3G	NP2				✓	N.D.*	✓
MCF-7	S3A	NP1	++		+++	✓	✓	N.D.
	S3F	NP1, NP2				NO	NO	N.D.
MDA-MB-468	S3A	NP1				NO	NO	N.D.
	S3F	NP1, NP2				NO	NO	N.D.

The effects of the expression of the semaphorins in the four tumor cell lines on tumor development, tumor angiogenesis and on the anchorage free growth of the cells are summarized. The relative expression levels of the relevant semaphorin binding receptors in each of the cell lines are shown as well (High level expression: +++, Low level expression: +). N.D., not determined.

* MDA-MB-231 derived tumors expressing sema3A and tumors derived from MDA-MB-435 expressing sema3F and sema3G did not develop and it was therefore not possible to measure effects on tumor angiogenesis.

** Estimation of the relative expression levels of plexD1 is based on estimation of mRNA levels.

*** Sema3F binds to np1 with a 10 fold lower affinity as compared to its affinity for np2 but it is unclear whether np1 can transduce sema3F signals.

To put this prediction to the test we expressed sema3A and sema3F in MDA-MB-468 breast cancer cells, which do not express np1, np2 or PlexD1 (Fig. 1D). These cells form slowly growing tumors in mammary fat pads of nu/nu balb/c mice. In agreement with our prediction, neither the expression of recombinant sema3A nor expression of sema3F significantly inhibited the formation of tumors from these cells (Fig. 5, E–F).

### The effects of different sema3s on tumor angiogenesis

**Figure 6:**
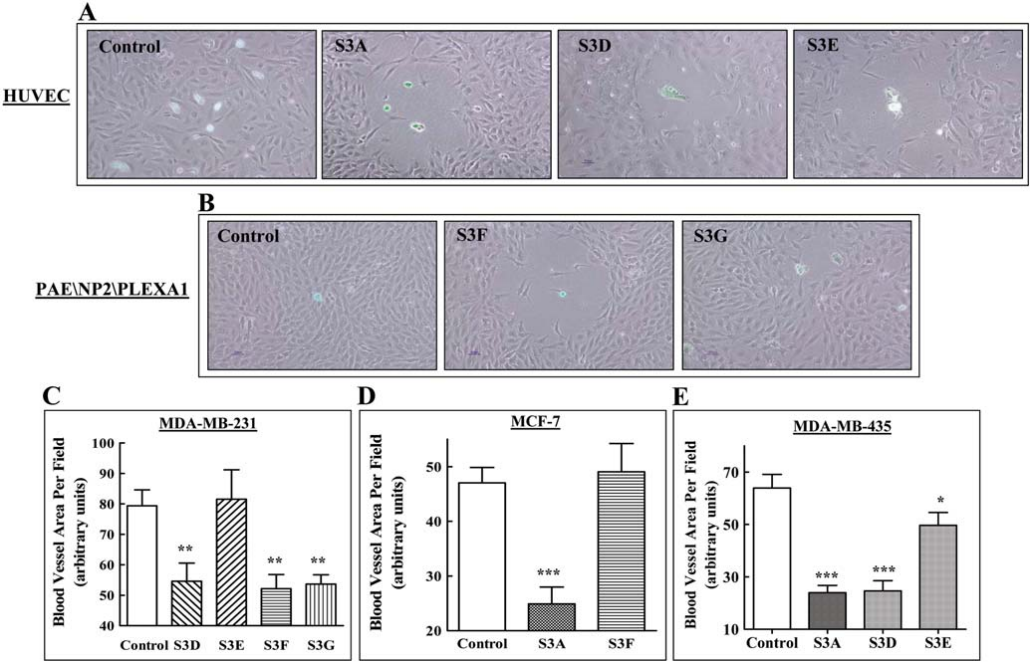
Different sema3s repel endothelial cells *in-vitro* and reduce the density of tumor associated blood vessels *in-vivo*. (A) Control HEK293 cells infected with an empty lentiviral vector or HEK293 cells expressing sema3A, sema3D or sema3E were seeded on top of a monolayer of HUVEC cells as described in materials and methods. The HEK293 cells were labeled with the fluorescent vital dye DIasp prior to seeding. Shown are composite pictures taken by phase and fluorescent microscopy. (B) Control HEK293 cells infected with an empty lentiviral vector or HEK293 cells expressing sema3F or sema3G were seeded on a monolayer of PAE cells expressing np2 and plexA1 as described in materials and methods. The HEK293 cells were stained with DIasp and photographed as described in material and methods (C) The average area of blood vessels per microscopic field was determined in sections derived from tumors that developed from MDA-MB-231 cells infected with empty lentiviruses or from MDA-MB-231 cells expressing different sema3s as described in materials and methods. Since the tumors that did develop from sema3A expressing cells were extremely small (Fig. 3) we could not determine the density of blood vessels in them. (D) The average area of blood vessels per microscopic field was determined in tumors derived from control MCF-7 cells or from MCF-7 cells expressing sema3A or sema3F as described in material and methods. (E) The average area of blood vessels per microscopic field was determined in tumors that developed from control MDA-MB-435 cells or from MDA-MB-435 cells expressing different sema3s as described in material and methods. No tumors developed from sema3G and sema3F expressing MDA-MB-435 cells (Fig. 4).

Sema3F was characterized in several studies as an inhibitor of tumor angiogenesis and as a repulsive factor for endothelial cells [15], [16], [28] and sema3A was also found to function as an inhibitor of VEGF induced angiogenesis and as a repulsive factor for endothelial cells although not as an inhibitor of tumor angiogenesis [17], [19], [29], [30]. To compare the repulsive properties of different sema3s we seeded HEK293 cells secreting different semaphorins on top of monolayers of human umbilical vein endothelial cells (HUVEC) at clonal densities. Control cells infected with empty vector did not repel endothelial cells while sema3A, sema3D and sema3E expressing cells repelled endothelial cells efficiently (Fig. 6A). However, the np2 agonists sema3F and in particular sema3G repelled HUVEC much less potently than the np1 agonists or the PlexD1 agonist sema3E, possibly because these cells contain about 3 fold less np2 as compared to np1 [5] (data not shown). We therefore seeded HEK293 cells expressing either sema3F or sema3G on top of porcine aortic endothelial (PAE) cells engineered to co-express recombinant np2 and plexA1 [15]. As expected, these cells were repelled very strongly by sema3F. Surprisingly they were repelled much less potently by sema3G suggesting that plexA1 may not be able to transduce sema3G signals (Fig. 6B).

To find out if the various sema3s inhibit tumor angiogenesis, we determined the concentration of blood vessels in tumors that developed from control or from sema3s expressing cells. Since sema3A inhibited tumor formation in MDA-MB-231 cell almost completely we could not determine the concentration of blood vessels in this case. However, expression of sema3D in MDA-MB-231 cells resulted in the formation of tumors containing a 40% lower density of blood vessels as compared to tumors that developed from control cells (Fig. 6C). The reduction in the concentration of tumor associated blood vessels was not correlated with the types of semaphorin receptors expressed by the cancer cells since expression of the np2 agonists sema3F and sema3G also reduced the concentration of blood vessels in tumors derived from MDA-MB-231 cells by about 40% (Fig. 6C) even though sema3G did not inhibit the development of tumors from these cells (Fig. 3, G–H). In-contrast, even though sema3E expression in MDA-MB-231 cells significantly inhibited the development of tumors (Fig. 3D) and even though sema3E expressing cells repulse HUVEC efficiently (Fig. 6A) the expression of sema3E in these cells did not reduce significantly the concentration of blood vessels in resulting tumors (Fig. 6C).

We also examined the effects of sema3A and sema3F expression on the concentration of tumor associated blood vessels in MCF-7 cells. These tumors develop in the mammary fat pads of the mice only in the presence of slow estrogen release pellets. Expression of sema3A in these cells significantly reduced the concentration of tumor associated blood vessels. However, expression of sema3F did not (Fig. 6D). The lack of inhibition in this case may perhaps be explained by findings suggesting that estrogen is an inhibitor of np2 expression [31].

In the case of tumors that develop from np2 expressing MDA-MB-435 cells, we found that the expression of sema3A and sema3D reduced the concentration of blood vessels in resulting tumors by 65% (Fig. 6E) even though tumor development from these cells was not inhibited at all by sema3A (Fig. 3B). It was not possible to determine the blood vessel concentration in tumors that developed from cells expressing sema3F or sema3G since the resulting tumors were too small or non-existent as in the case of sema3G. Expression of sema3E in MDA-MB-435 cells produced a small but significant 28% decrease in the concentration of blood vessels in resulting tumors even though tumor formation from these cells was not inhibited by sema3E (Fig. 4D).

Taken together, these experiments indicate that although most of the semaphorins are able to inhibit tumor angiogenesis as manifested by the reduction in the concentration of blood vessels in tumors in response to the expression of recombinant semaphorins. However, even though the inhibition may contribute to the inhibition of tumor progression, there was generally no correlation between the effects of the sema3s on tumor angiogenesis and their effect on tumor development (Table 1).

### The effects of different sema3s on the behavior of the tumor cells in-vitro

The experiments described in the previous sections suggest that semaphorin expression should modulate the behavior of tumor cells. Indeed, sema3s such as sema3F and sema3B were reported to inhibit the adhesion, spreading and proliferation of various types of tumor cells [13], [16], [18]. However, the proliferation of the tumor cell types which we have used in the present study was not inhibited as a result of the expression of the various semaphorins when the tumor cells were grown in tissue culture dishes (data not shown). We also examined the effect of the expression of the different semaphorins on the adhesion of the various tumor cells to plastic or to fibronectin. However, none of these semaphorins affected the adhesion of the tumor cells regardless of whether the substrate was plastic or fibronectin (data not shown).

**Figure 7:**
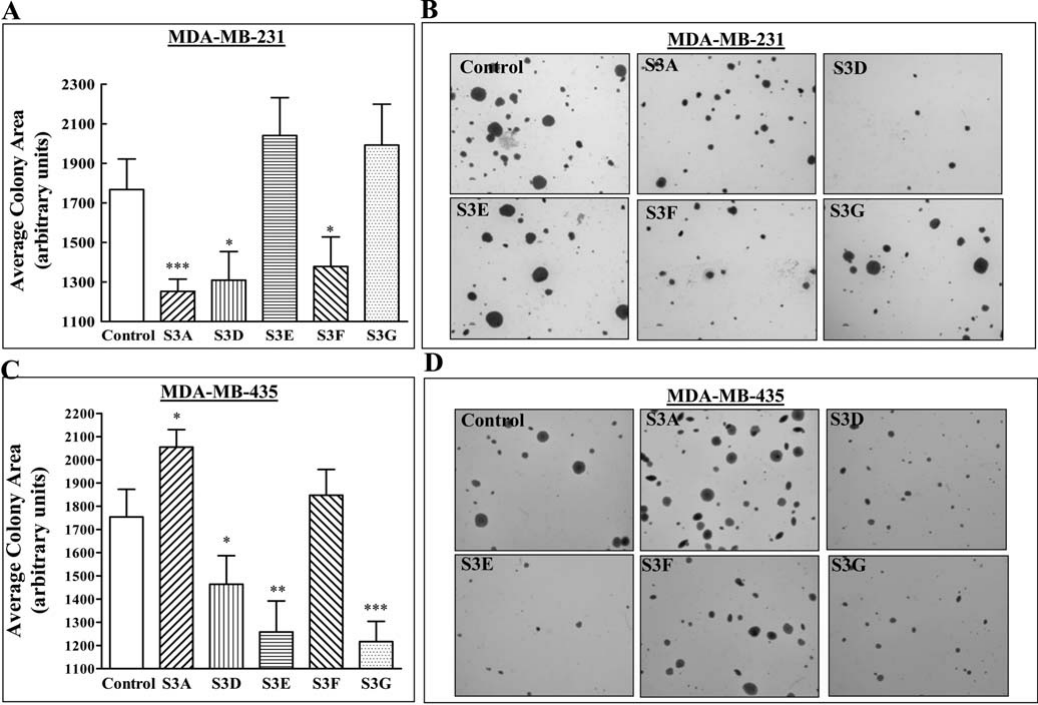
Different sema3s inhibit the formation of soft agar colonies from MDA-MB-231 or MDA-MB-435 cells. (A) Single cell suspensions of control MDA-MB-231 cells or MDA-MB-231 cells expressing different sema3s were seeded in soft agar as described in materials and methods. Colonies were allowed to form for 21 days. The colonies were then stained with crystal violet and microscopic fields photographed. The average colony area of colonies with a diameter exceeding 150 µm was then determined as described in materials and methods. (B) Photographs of representative microscopic fields containing crystal violet stained colonies that developed in soft agar from control MDA-MB-231 cells or from MDA-MB-231 cells expressing the indicated sema3s. (C) The formation of colonies in soft agar from control MDA-MB-435 cells or from MDA-MB-435 cells expressing the indicated sema3s was determined as described in materials and methods. (D) Photographs of representative microscopic fields containing crystal violet stained colonies that developed in soft agar from control MDA-MB-435 cells or from sema3s expressing MDA-MB-435 cells.

The ability to form colonies in soft-agar is a hallmark that differentiates many types of cancer cells from their normal counterparts [32]–[34]. We therefore determined if the expression of different class-3 semaphorins in MDA-MB-231 or MDA-MB-435 cells affects their ability to form colonies in soft-agar. None of the semaphorins inhibited completely the formation of colonies by MDA-MB-231 cells. However, the expression of sema3A and sema3D, semaphorins that strongly inhibited tumor formation from these cells (Fig. 3), also inhibited significantly the formation of large colonies in soft agar (Fig. 7, A–B). Surprisingly, expression of sema3F also inhibited significantly the formation of large colonies in soft agar despite the absence of np2 receptors on these cells. However, the expression levels of sema3F were the highest of all the semaphorins we examined (Fig. 2A) and sema3F was also able to inhibit the formation of tumors from these cells (Fig. 3B). Sema3F binds to np1, albeit with a 10 fold lower affinity as compared to its binding affinity to np2 [35] and there is one additional report suggesting that it may also utilize np1 for signal transduction [36]. It is therefore possible that this inhibitory effect is mediated by np1. Another np2 agonist, sema3G, which in contrast with sema3F does not inhibit the development of tumors from MDA-MB-231 cells (Fig. 3H) and does not bind to np1 [37], had no effect on the development of colonies from these cells (Fig. 7A). MDA-MB-231 cells also express the sema3E receptor PlexD1 and expression of sema3E inhibits the formation of tumors from these cells (Fig. 3D). However, sema3E failed to inhibit the formation of colonies from MDA-MB-231 cells (Fig. 7, A–B).

We also determined whether sema3D, sema3F and sema3G, which inhibit tumor formation from MDA-MB-435 cells (Fig. 4), also inhibit the anchorage free growth of these cells. Both sema3D and sema3G inhibited colony formation efficiently as expected. However, to our surprise we found that sema3F did not, even though it did inhibit almost completely the formation of tumors from these cells (Fig. 7, C–D). Another unexpected observation was that sema3E, which did not inhibit the formation of tumors from these cells was able to inhibit colony formation (Fig. 7, C–D). Lastly, we expected that sema3A will not affect colony formation since its receptor is not expressed by MDA-MB-435 cells (Fig. 1). Surprisingly, we found that not only was colony formation not inhibited but it was even significantly enhanced (Fig. 7, C–D). Taken together our results suggest that despite a number of exceptions, in most cases inhibition of tumor growth by sema3s is correlated with their ability to inhibit the formation of soft agar colonies from the tumor cells (Table 1).

### Enhancement of tumor development from MDA-MB-435 cells by np1 expression is inhibited by co-expression of sema3A

**Figure 8:**
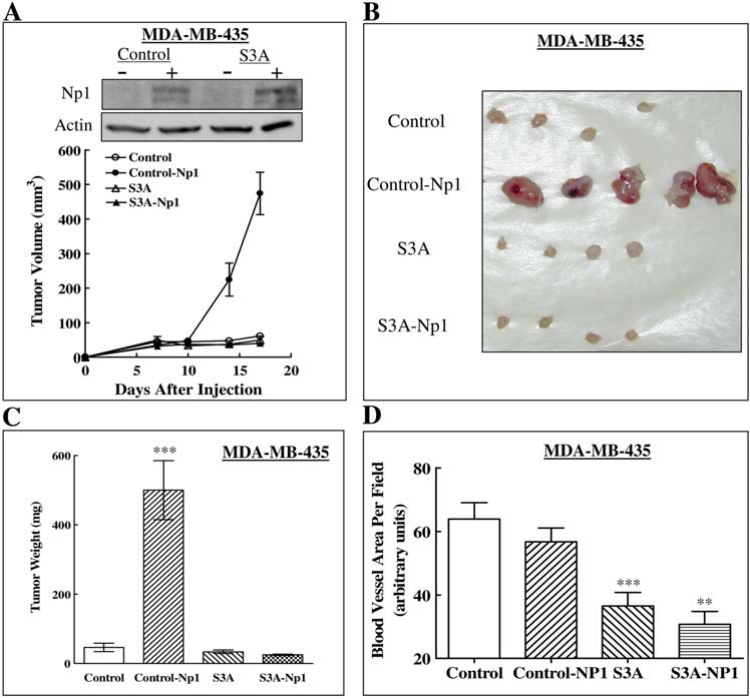
Expression of np1 in MDA-MB-435 cells enhances the growth of resulting tumors and sema3A abrogate the enhancing effect. (A) A western blot comparing the expression of np1 in MDA-MB-435 cells infected with lentivirus directing expression of np1, sema3A or an empty vector is shown at the top. The average volumes of the tumors that developed following the implantation of these cells in mammary fat pads of nude mice as a function of time after implantation is shown in the lower part. (B) Photographs of tumors excised at the end of the experiment. (C) The average weight of the tumors at the end of the experiment was determined as described in materials and methods. (D) The average area of blood vessels/microscopic field in tumor sections was determined as described in materials and methods.

The experiments described above suggest that the expression of specific sema3s receptors by tumor cells is probably the most important factor that determines whether a given sema3 will function as an inhibitor of tumor development. To test this hypothesis further we expressed np1 in MDA-MB-435 cells in order to determine whether this would render tumors that develop from these cells sensitive to sema3A. The tumors that developed from MDA-MB-435 cells expressing np1 grew very rapidly to a much larger size than tumors derived from empty vector infected MDA-MB-435 cells following an initial lag (Fig. 8, A–C). Interestingly, the density of blood vessels within these tumors was not significantly different from that of control tumors (Fig. 8D). When the np1 agonist sema3A was co-expressed in these cells along with np1, the cells that expressed both genes reverted to the behavior exhibited by the control cells and formed slowly developing tumors thereby eliminating the growth advantage conferred by the presence of np1 (Fig. 8, A–C), but not that conferred by the presence of np2 which can be further inhibited by np2 agonists such as sema3F or sema3G (Fig. 4). Interestingly, the density of blood vessels in tumors that developed from MDA-MB-435 cells expressing sema3A or sema3A/np1 was similar and about 50% lower than the concentration of blood vessels in tumors that developed from control cells (Fig. 8D). These results also suggest independently that inhibition of angiogenesis may represent part of the mechanism by which semaphorins modulate tumor progression, but that it may not always be sufficient to inhibit tumor growth.

## Discussion

The identification of sema3B and sema3F as tumor suppressors [13], [14], [38], and the identification of sema3F and sema3A as inhibitors of angiogenesis [15], [39], suggested that additional sema3s may also possess anti-tumorigenic and anti-angiogenic properties. We show here for the first time that sema3A, sema3D, sema3E and sema3G display anti-tumorigenic properties. Furthermore, we show that all of these class-3 semaphorins can also inhibit tumor angiogenesis.

Many tumorigenic cell lines, including the cell lines we used, express different combinations of neuropilins and plexins [40]. Neuropilins as well as several types of plexins are also expressed in endothelial cells [19] and in some bone marrow derived cells [41] which are frequently recruited into the tumor microenvironment. Complexes formed between the neuropilins and several types of plexins mediate sema3s induced signal transduction with the exception of sema3E which signal through PlexD1 independently of neuropilins [2]. Our initial experiments suggested that the expression of given semaphorins in tumor cells sometimes inhibited and sometimes did not inhibit the development of tumors from different types of tumor cells.

To better understand these seemingly conflicting results we compared the effects of the expression of several semaphorins on the development of tumors from tumorigenic cell lines differing in their expression pattern of semaphorin receptors so as to find out if we can identify a property that can predict whether a given semaphorin will be able to inhibit the development of tumors from a given type of tumor cell. The expression of the different recombinant semaphorins in the tumor cells did not change significantly the expression of the primary semaphorin binding receptors np1, np2 and PlexD1 in the tumor cells. Therefore, observed differences in responses to the expression of different semaphorins in the tumor cells were not due to semaphorin induced changes in the expression of direct semaphorin receptors. Semaphorin signaling through these receptors may also be modulated by changes in the expression levels of endogenous semaphorins and by changes in the expression and activity of neuropilin associated receptors such as additional types of plexins and adhesion receptors known to modulate semaphorin signal transduction [2] which are present on tumor cells as well as on tumor associated stromal cells such as endothelial cells. We have not tested these parameters systematically due to the lack of appropriate specific antibodies directed against these different proteins and because of the large volume of assays required to monitor such changes systematically.

Regardless of these possible modulating influences, we have found that the expression of a semaphorin receptor able to bind the specific recombinant semaphorin we expressed in the tumor cells was the property that correlated best with the ability to successfully inhibit tumor development (Table 1). There were only two cases in which this correlation did not successfully predict whether a specific semaphorin will be able to inhibit tumor development from a given type of tumor cell. In the first such example Sema3E was not able to inhibit tumor development from MDA-MB-435 cells. However, the expression levels of the mRNA encoding the sema3E receptor plexD1 in the MDA-MB-435 cells are lower than their levels in MDA-MB-231 cells which may perhaps account for the discrepancy. The second example was the successful inhibition by sema3F of tumor formation from MDA-MB-231 cells which express np1 but not np2 and thus should not have been inhibited by sema3F. However, the concentration of np1 in these cells is relatively high [4]. Sema3F is known to bind to np1 with a 10 fold lower affinity as compared to np2. Even though sema3F is usually viewed a pure np2 agonist there is nevertheless some evidence suggesting that sema3F may be able to transduce signals using np1 [36]. It is thus possible that the inhibition of tumor development from MDA-MB-231 cells by sema3F is mediated by np1 and that it is augmented by the relatively high expression level obtained with sema3F and by the anti-angiogenic effect displayed by sema3F in tumors derived from this cell type. The lack of an anti-tumorigenic effect of sema3F in tumors developing from sema3F expressing MCF-7 cells which also express np1 but no np2 may be explained by the lower concentration of np1 receptors in these cells and by the absence of a sema3F induced anti-angiogenic effect which is probably the result of np2 down regulation in endothelial cells of blood vessels due to the effects of prolonged estrogen administration [31].

In order to determine whether there is a correlation between the anti-angiogenic activity of specific semaphorins and their ability to inhibit tumor development we measured the effects of the expression of several semaphorins in several tumor cell types on the concentration to tumor associated blood vessels. We have presented here for the first time evidence indicating that sema3D, sema3G sema3E and sema3A can significantly reduce the concentration of microvessels in tumors that develop from tumor cells that express these semaphorins. Surprisingly, we found that reduction in the concentration of tumor associated blood vessels was frequently not correlated with the anti-tumorigenic effect of given semaphorins. For example, even though sema3G and sema3A expression did not inhibit at all the development of tumors from MDA-MB-231 and MDA-MB-435 cells respectively, they nevertheless strongly reduced the concentration of tumor associated blood vessels. These observations indicates that inhibition of tumor angiogenesis by the sema3s we examined was probably not sufficiently effective to affect tumor growth. Anti-VEGF antibodies were reported to reduce the concentration of blood vessels in MDA-MB-231 derived tumors by as much as 70% [42], [43] while individual sema3s reduced blood vessel densities in such tumors by up to 40%. It is of course rather difficult to compare two different studies in which the methods used to evaluate blood vessel density were not identical. Nevertheless, it is likely that the anti-angiogenic effects of individual sema3s were not sufficiently strong so as to enable inhibition of tumor development in the case of the cancer cells that we examined. It is however likely that the anti-angiogenic effects of the semaphorins will assume more importance in the case of rapidly growing tumors that may be more dependent on efficient angiogenesis than slowly growing tumors. We have previously shown that combinations of sema3s that interact with different semaphorin receptors can inhibit the proliferation of endothelial cells more effectively than individual sema3s [19]. Our results suggest that combinations of such sema3s may perhaps be able to increase the anti-angiogenic effects to the point at which they may affect tumor development more effectively.

Another parameter we have examined as a possible predictor for the effectiveness of class-3 semaphorins as anti-tumorigenic agents was effects of class-3 semaphorin expression in tumor cells on the behavior of the tumor cells *in-vitro*. Contrary to previous reports which observed sema3F and sema3A induced changes in adhesion of tumor cells to fibronectin coated dishes [16], [18], [44], we could not see any effects of any of the semaphorins we tested on the adhesion to fibronectin of any of the tumor cells used here. We also could not detect any effects of semaphorin expression in the various tumor cells on the proliferation of the tumor cells in regular 2D cell culture. This observation contrasts with the strong effects observed when using cultured endothelial cells [19]. However, some of the semaphorins we used inhibited the growth of colonies of tumor cells in soft agar. The correlation between the ability to inhibit the growth of colonies in soft-agar and the anti-tumorigenic effects of the semaphorins was better than the correlation with the anti-angiogenic effects of the semaphorins, but it was nevertheless a less reliable predictor for the effectiveness of the semaphorins as anti-tumorigenic agents as compared with the presence of appropriate semaphorin receptors in the tumor cells. For example, sema3F did not inhibit the formation of colonies from MDA-MB-435 cells despite the presence of np2 receptors in these cells even though it inhibited strongly tumor formation. In contrast, sema3E inhibited strongly the formation of soft-agar colonies from MDA-MB-435 cells but did not inhibit the formation of tumors. Nevertheless, in most cases the ability to inhibit the formation of colonies in soft agar was correlated with the ability to inhibit the development of tumors *in-vivo*.

The presence of appropriate signaling semaphorin receptors on tumor cells does not necessarily imply that the anti-tumorigenic effects observed are due to direct effects on the tumor cells, even though in some cases that may be the case. There is some evidence indicating that neuropilins may be able to associate with receptors present on adjacent cells “in-trans” [45] and it is also possible that the final outcome *in-vivo* will depend on the effects of secondary effectors synthesized in response to semaphorins in responsive tumor cells, resulting in different responses *in-vivo* as compared to *in-vitro* experiments in which the only cell type is the tumorigenic cell. An example for such a modulator of semaphorin function is provided by the furin like pro-protein convertases, which are strongly up-regulated in cancer cells [46]. The furins cleave class-3 semaphorins at a conserved site and the cleavage results in inactive products in the case of sema3A and sema3B [23], [47]. In the case of sema3E the cleavage generates a pro-metastatic product that affects primarily endothelial cells rather than tumor cells [24] and is thus an example for an effect that will be seen only *in-vivo* but will not affect *in-vitro* assays such as the soft-agar colony formation assay. It should be noted that in our experiments the maximal amount of cleavage by pro-protein convertases never exceeded 15% of the total amount of sema3s found in the conditioned medium of producing tumor cells, and in the case of sema3E the cleavage was almost undetectable in both MDA-MB-231 and MDA-MB-435 cells, suggesting that the inhibitory effects that we observed are due to the effects of full length sema3E.

In conclusion, we have found for the first time that the semaphorins sema3A, sema3D, sema3E and sema3G possess anti-tumorigenic and anti-angiogenic properties similar to those displayed by the previously identified tumor suppressor sema3F. However, the anti-angiogenic effects are probably not sufficiently potent so as to enable inhibition of tumor development. The anti-tumorigenic effect of sema3s seems to be associated with the expression of appropriate sema3s receptors by the tumor cells although it is not clear if all the anti-tumorigenic effects are due to direct effects on the tumor cells. Our results argue that for maximal effectiveness, the selection of specific semaphorins or semaphorin combinations will have to take into account the identity of the semaphorin receptors expressed by the tumorigenic cells within target tumors.

## Supporting Information

Figure S1 The endogenous expression levels of NP-1, NP-2 and PlexD1 in MDA-MB-231 and MDA-MB-435 cells infected with lentiviruses directing the expression of different class-3 semaphorins. Cell lysates were prepared from MDA-MB-231 (panel A) and MDA-MB-435 (panel-B) cells infected with lentiviruses directing the expression of the indicated class-3 semaphorins or an empty lentiviral expression vector. The expression levels of NP-1 (Aa, Ba) and NP-2 (Ab, Bb) in the respective cell types were compared using western blot analysis as described in materials and methods. The expression of Plex-D1 (Ac, Bc) was detected by RT-PCR as described in Fig. 1.
(6.76 MB TIF)

Figure S2 The endogenous expression of NP-1, NP-2 and Plexin-D1 in MCF-7 and MDA-MB-468 infected with lentiviruses directing expression of sema3A or sema3F. Cell lysates were prepared from MCF-7 (panel-A) or MDA-MB-468 (panel-B) cells infected with control lentiviruses or lentiviruses directing expression of sema3A or sema3F. The expression of NP-1 (Aa, Ba) and NP-2 (Ab, Ba) was detected using western blot analysis as described in materials and methods. The expression levels of the Plex-D1 mRNA in the two cell types (Ac, Bb) was detected by RT-PCR as described in materials and methods.
(4.79 MB TIF)

Figure S3 Photographs of excised tumors derived from MDA-MB-231 cells expressing sema3A, sema3F, sema3E and an empty expression vector. Control MDA-MB-231 cells infected with empty lentiviruses (C) or MDA-MB-231 cells expressing recombinant sema3A (S3A) sema3F (S3F) or sema3E (S3E) were implanted in the mammary fat pads of balb\c nu/nu mice as described. At the end of the experiment tumors were excised and photographed.
(4.93 MB TIF)
