# Supplementary figures and images for: Successful Inhibition of Tumor Development by Specific Class-3 Semaphorins Is Associated with Expression of Appropriate Semaphorin Receptors by Tumor Cells
Source: PLoS One. 2008 Sep 26;3(9):e3287. doi: 10.1371/journal.pone.0003287 (PMC2538586; doi:10.1371/journal.pone.0003287)

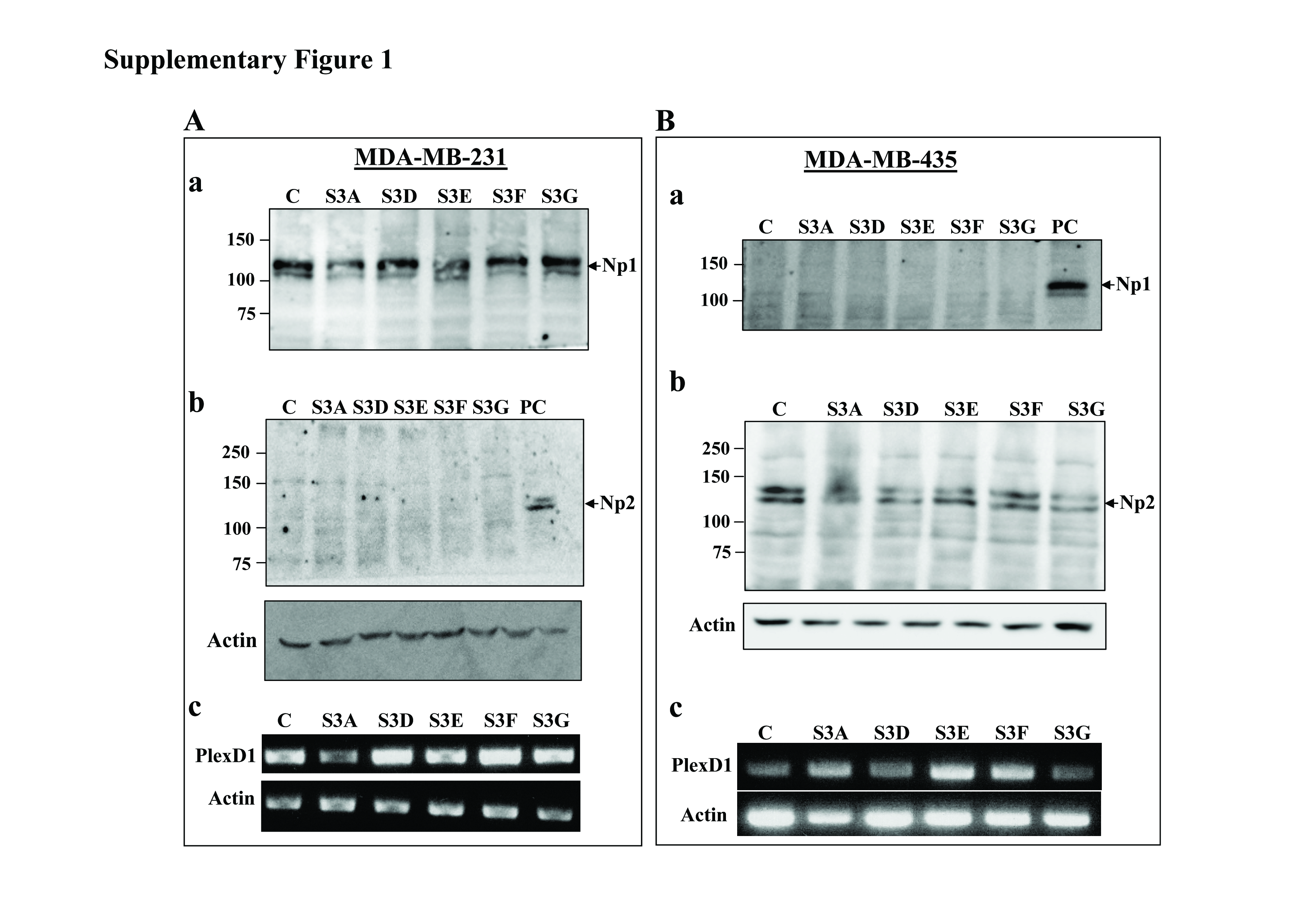

Supplement: Figure S1 — The endogenous expression levels of NP-1, NP-2 and PlexD1 in MDA-MB-231 and MDA-MB-435 cells infected with lentiviruses directing the expression of different class-3 semaphorins. Cell lysates were prepared from MDA-MB-231 (panel A) and MDA-MB-435 (panel-B) cells infected with lentiviruses directing the expression of the indicated class-3 semaphorins or an empty lentiviral expression vector. The expression levels of NP-1 (Aa, Ba) and NP-2 (Ab, Bb) in the respective cell types were compared using western blot analysis as described in materials and methods. The expression of Plex-D1 (Ac, Bc) was detected by RT-PCR as described in Fig. 1. (6.76 MB TIF) [file pone.0003287.s001.tif]

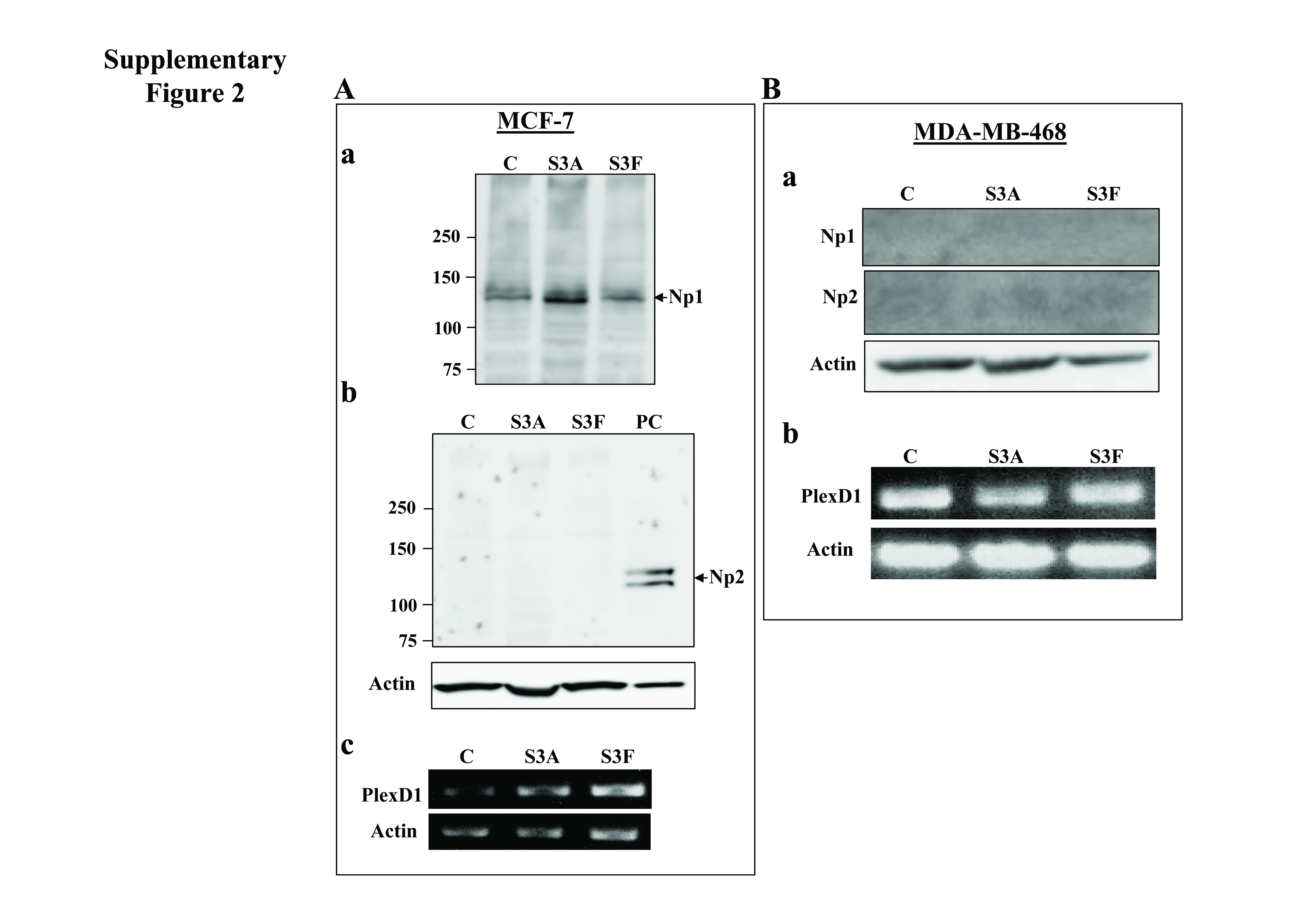

Supplement: Figure S2 — The endogenous expression of NP-1, NP-2 and Plexin-D1 in MCF-7 and MDA-MB-468 infected with lentiviruses directing expression of sema3A or sema3F. Cell lysates were prepared from MCF-7 (panel-A) or MDA-MB-468 (panel-B) cells infected with control lentiviruses or lentiviruses directing expression of sema3A or sema3F. The expression of NP-1 (Aa, Ba) and NP-2 (Ab, Ba) was detected using western blot analysis as described in materials and methods. The expression levels of the Plex-D1 mRNA in the two cell types (Ac, Bb) was detected by RT-PCR as described in materials and methods. (4.79 MB TIF) [file pone.0003287.s002.tif]

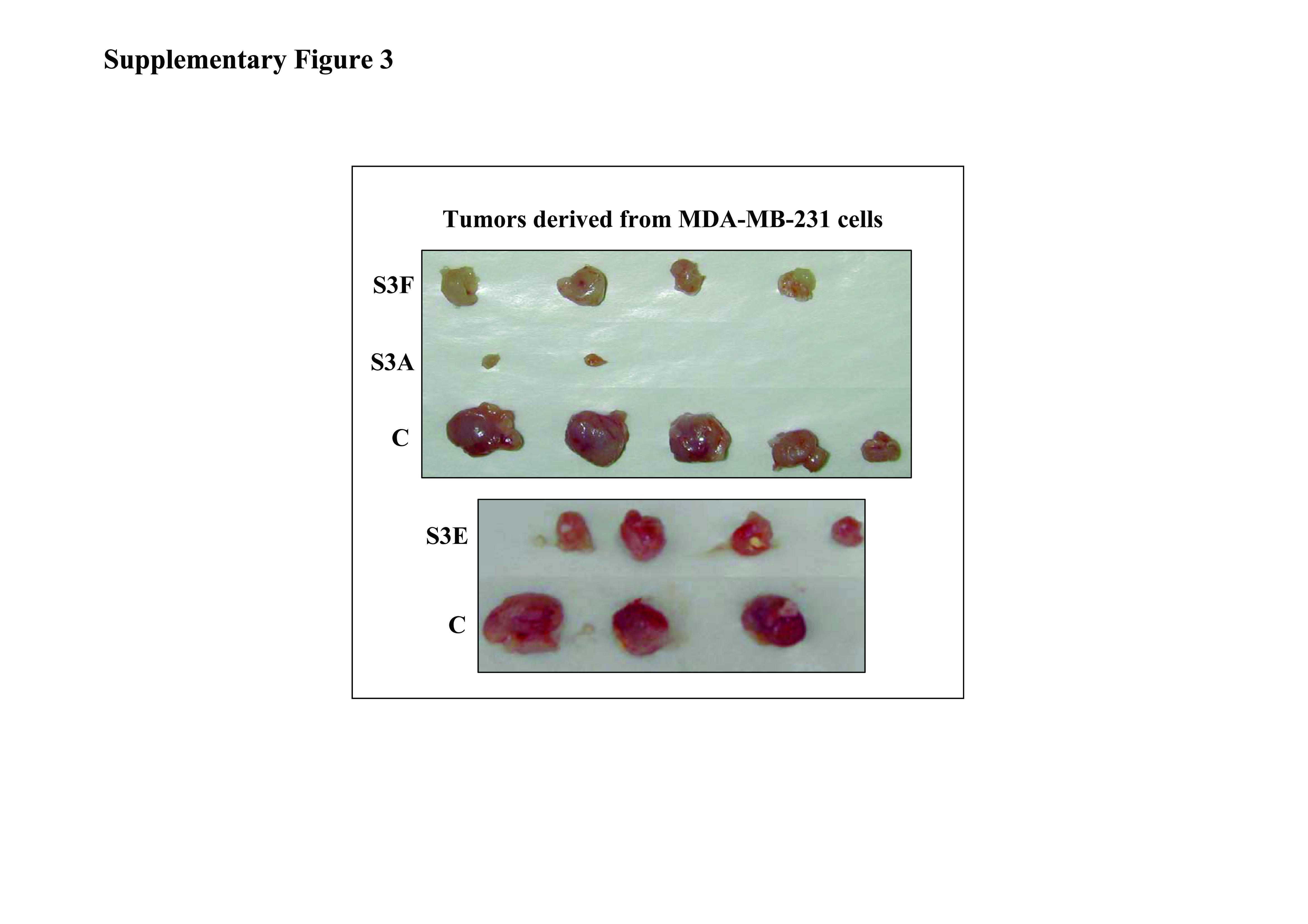

Supplement: Figure S3 — Photographs of excised tumors derived from MDA-MB-231 cells expressing sema3A, sema3F, sema3E and an empty expression vector. Control MDA-MB-231 cells infected with empty lentiviruses (C) or MDA-MB-231 cells expressing recombinant sema3A (S3A) sema3F (S3F) or sema3E (S3E) were implanted in the mammary fat pads of balb\c nu/nu mice as described. At the end of the experiment tumors were excised and photographed. (4.93 MB TIF) [file pone.0003287.s003.tif]
